# Feasibility Study on Prenatal Cardiac Screening Using Four-Dimensional Ultrasound with Spatiotemporal Image Correlation: A Multicenter Study

**DOI:** 10.1371/journal.pone.0157477

**Published:** 2016-06-17

**Authors:** Liqing Zhao, Yurong Wu, Sun Chen, Yunyun Ren, Ping Chen, Jianmei Niu, Cao Li, Kun Sun

**Affiliations:** 1 Division of Pediatric Cardiology, Xin Hua Hospital Affiliated to Shanghai Jiao Tong University School of Medicine, Shanghai, People's Republic of China; 2 Department of medical ultrasound, The Obstetrics & Gynecology Hospital of Fudan University, Shanghai, People's Republic of China; 3 Department of medical ultrasound, Shanghai First Maternity and Infant Hospital, Shanghai, People's Republic of China; 4 Department of medical ultrasound, The International Peace Maternity & Child Health Hospital of China welfare institute, Shanghai, People's Republic of China; University of Barcelona, SPAIN

## Abstract

**Objective:**

This study aimed at investigating the feasibility of using the spatiotemporal image correlation (STIC) technology for prenatal cardiac screening, finding factors that influence the offline evaluation of reconstructed fetal heart, and establishing an optimal acquisition scheme.

**Methods:**

The study included 452 gravidae presenting for routine screening at 3 maternity centers at 20–38 gestational weeks. The factors influencing the quality of STIC volume data were evaluated using *t* test, chi-square test, and logistic regression analysis. The predictive power was evaluated using the receiver operating characteristic (ROC) curve.

**Results:**

Among the 452 fetuses enrolled, 353 (78.1%) were identified as successful and 99 (21.9%) as failure of evaluation of the reconstructed fetal heart. The total success rate of qualified STIC images was 78.1%. The display rates of reconstructed cardiac views were 86.5% (four-chamber view), 92.5% (left ventricular outflow tract view), 92.7% (right ventricular outflow tract view), 89.9% (three-vessel trachea view), 63.9% (aortic arch view), 81.4% (ductal arch view), 81% (short-axis view of great vessels), 80.1% (long-cava view), and 86.9% (abdominal view). A logistic regression analysis showed that more than 28 gestational weeks [OR = 0.39 (CI 95% 0.16, 0.19), *P* = 0.035], frequent fetal movements [OR = 0.37 (CI 95% 0.16, 0.87), *P* = 0.022], shadowing [OR = 0.36 (CI 95% 0.19, 0.72), *P* = 0.004], spine location at 10–2 o’clock [OR = 0.08 (CI 95% 0.02, 0.27), *P* = 0.0], and original cardiac view [OR = 0.51 (0.25, 0.89), *P* = 0.019] had a significant impact on the quality of STIC. The area under the ROC curve was 0.775.

**Conclusions:**

Fetal cardiac-STIC seems a feasible tool for prenatal screening of congenital heart diseases. The influence factors on the quality of STIC images included the intensity of training, gestational age, fetal conditions and parameter settings. The optimal acquisition scheme may improve the application and widespread use of cardiac STIC.

## Introduction

An accurate antenatal diagnosis of congenital heart disease (CHD) is important for an appropriate prenatal counseling, which could involve considerable planning for the location and time of delivery and optimal perinatal care [1, 2]. Postnatal outcomes of certain types of CHD have been shown to improve with the help of prenatal diagnosis [2–3]. Most cardiac lesions can be detected using fetal echocardiography performed by experienced experts [4–5]; however, the number of experts familiar with fetal echocardiography is particularly small, considering the large population of high-risk pregnancies in China.

The World Society of Ultrasound in Obstetrics and Gynecology has proposed that the left ventricular outflow tract (LVOT) view, right ventricular outflow tract (RVOT) view, and three-vessel trachea view (3VV) should also be necessary views for fetal cardiac screening in addition to the four-chamber view (4CV) [6]. Although the recommended screening views were normalized in Shanghai, problems still exist in the following aspects: (1) some kinds of CHD can still be overlooked; (2) high-risk pregnancies can take a long time before being referred to a fetal cardiologist to accomplish fetal echocardiography; and (3) pregnancies with complicated fetal cardiac malformations can hardly receive exact diagnosis and proper counseling in time.

The accuracy of the antenatal diagnosis of CHD has improved with the development of a better image quality and the introduction of a new technology such as the spatiotemporal image correlation (STIC) technology. He et al reported a high sensitivity (97.4%) and specificity (99.6%) using the STIC technology combined with two-dimensional (2D) ultrasound for a definite diagnosis of fetal heart malformations [7]. Yagel et al provided a 6.6% added value in achieving, or honing or enhancing diagnosis [8]. STIC is an automated volume acquisition technology that allows information on fetal hearts to be stored in 4D cine loop sequences [9]. Moreover, the acquired STIC volume data can be reviewed offline even in remote centers [10]. When the STIC volume data are acquired, any reconstructed plane of the fetal heart can be theoretically reviewed. This approach can possibly reduce some dependence on the need for experience and skills on fetal echocardiography, which means sonographers who have mastered the skill of STIC can evaluate some unobtainable views from the acquired volume data instead of searching for the views for a long time. With the help of offline analysis of STIC, the aforementioned problems may be solved through proper communication between obstetricians and fetal cardiologists. However, the present study has not performed a large multicenter data study on the use of STIC in prenatal screening or diagnosis since the introduction of the technology in 2003 [11].

The current study aimed at investigating the possibility of using STIC technology by obstetric sonographers for prenatal cardiac screening, finding factors that influence on the offline evaluation of reconstructed cardiac views, and establishing an optimal guide for acquisition.

## Materials and Methods

The current study was a blind study carried out using a multicenter approach. Sonographers were united from three tertiary obstetrics and gynecology centers in Shanghai [The International Peace Maternity & Child Health Hospital of China Welfare Institute (center A), the Obstetrics & Gynecology Hospital of Fudan University (center B), and the Shanghai First Maternity and Infant Hospital (center C)] to acquire STIC volume data and transfer these data to the fetal cardiology unit. The present study employed an obstetric–cardiology closely connected mode in the cardiac screening, which enabled obstetric sonographers to acquire cardiac STIC volume data during the targeted organ scans, and send high-risk or suspicious malformation data to fetal heart unit in time. In this study, the focus was on data acquisition, transmission, and analysis depending on this connected mode.

Before the study, a training was organized focusing on the study implementation and operation of STIC data acquisition, storage, and transmission. An application specialist from the GE company and an experienced fetal cardiologist familiar with the STIC technology took charge of the training. The training was combined with the form of lecture and hands-on. Unfortunately, for some reason, the sonographers from center C missed the training and received the relevant materials later by email and were defined as having received “insufficient training.” The same materials were also emailed to the other two centers, A and B, after the training.

### Machine and patients

The STIC volume data were acquired using a Voluson E8 machine (GE Healthcare Ultrasound, WI, USA) with a 4–8 MHz transabdominal probe during a routine screening. The ultrasound examination was performed using the ALARA (as low as reasonably achievable) principle. The pregnancies enrolled in this study were a mixed group of low- and high-risk pregnancies referred to the three centers A, B, and C for targeted organ scans or following scans from 20 to 38 gestational weeks (GWs). Inclusion criteria for this study were as follows: singleton fetuses that have passed the cardiac screening in the target organ scans, with normal amniotic fluid, with no known aneuploidy, and without fetal arrhythmia. The research was approved by the XinHua Hospital Ethics Committee (Approval Number: XHEC-C-2014-053). All patients provided verbal informed consent to participate before undergoing STIC imaging. This consent procedure was approved by the XinHua Hospital Ethics Committee.

### Data acquisition

To confirm the gestational age (GA), all included women had undergone first-trimester fetal crown-rump length measurement. Biparietal diameter, femur length, abdominal circumference, and head circumference were also measured to check the GA again on the day of the examination. Basic information was recorded during the examination concerning maternal age, pre-gestational weight, height, reproductive history, etc.

During acquisition, the sonographers addressed the conditions with respect to fetal movements, shadowing of bones or limbs, and location of the spine. No more than five STIC attempts were performed on one fetus, and the patients were given a rest if they had an unsuitable fetal position or frequent fetal movements before another attempt was made. A recommended scheme about acquisition parameters was offered according to the experiences from the heart center and other studies [12] through the training. Only gray-scale imaging was performed, and the 2D image quality was optimized before STIC acquisition. A 4CV was a “necessary original view” to acquire the volume data; the 3VV and the long-axis view of the aortic arch (AA) were “selective original views.” The volume angle ranged from 15° to 40° and the acquisition time from 7.5 to 15 s irrespective of specific GA. The value of the volume angle was more than the GW, while the acquisition time was not limited according to the training.

### Data analysis

The STIC volume data were analyzed by two operators familiar with fetal cardiology using the Voluson 4DView 10.5 postprocessing software. These two operators were blind to the characteristics of enrolled pregnancies. The image number of each fetus (volume numbers), original cardiac views included (only 4CV or more), acquisition time, volume angle, and region-of-interest (ROI) setting were recorded. The ROI was recorded in the form of the structures encompassed in a trapezoid box, including four chambers, transverse section of descending aorta (DAO), spine, and limbs. Adequate ROI was defined as encompassing at least 4CV, DAO, and part of the spine from the recommended scheme of the present study. As mentioned earlier, the value of the volume angle larger than the value of GA was recommended and their difference value was chosen (volume angle minus GA) as a statistical parameter.

**Fig 1:**
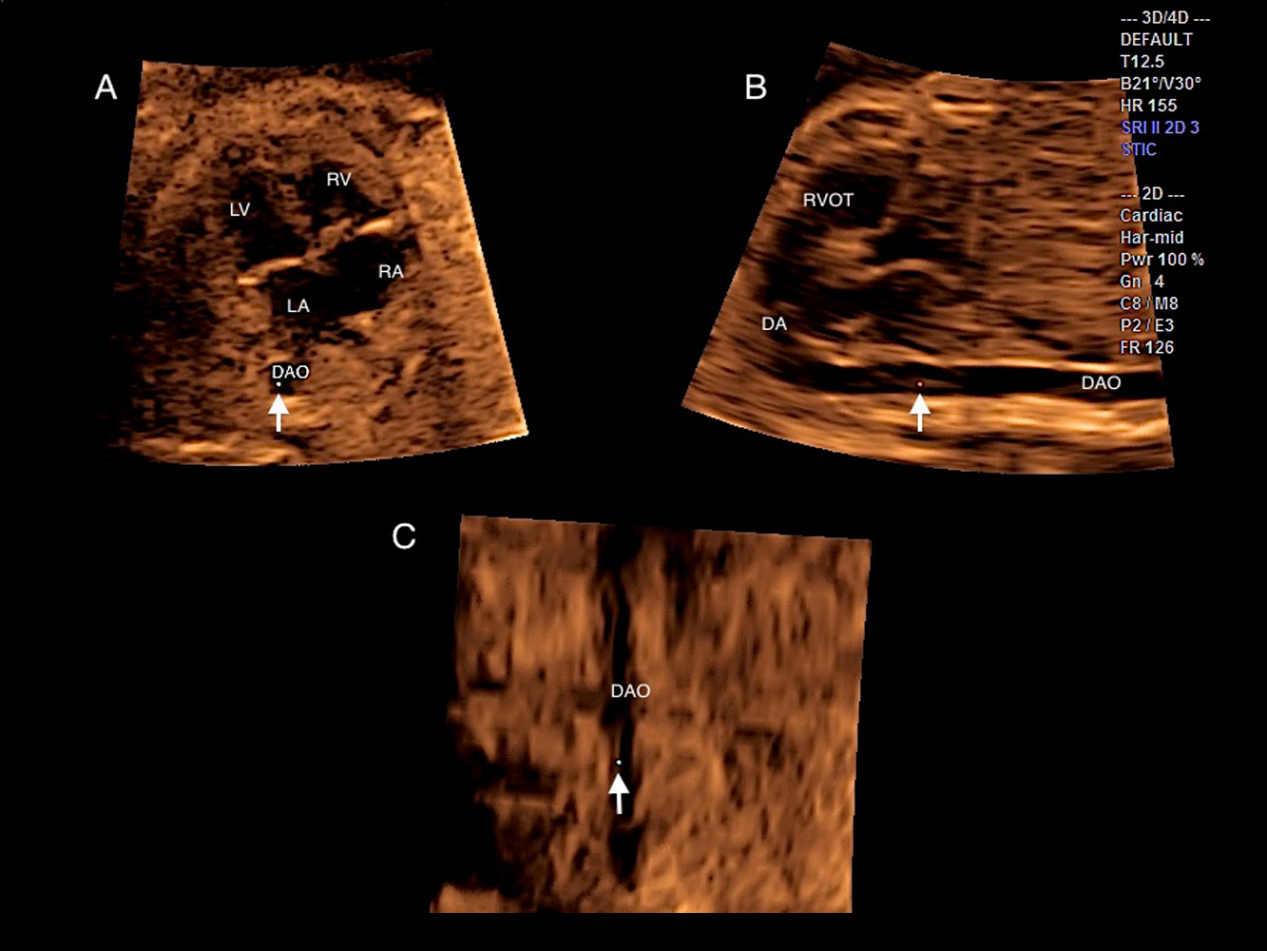
Multiplanar mode (4CV as the original acquisition plane): (A) 4CV; (B) DA; (C) transverse section of plane A. LV, left ventricle; RV, right ventricle; LA, left atrium; RA, right atrium; DAO, descending aorta; RVOT, right ventricular outflow tract; 4CV, four-chamber view; DA, ductal arch.

**Fig 2:**
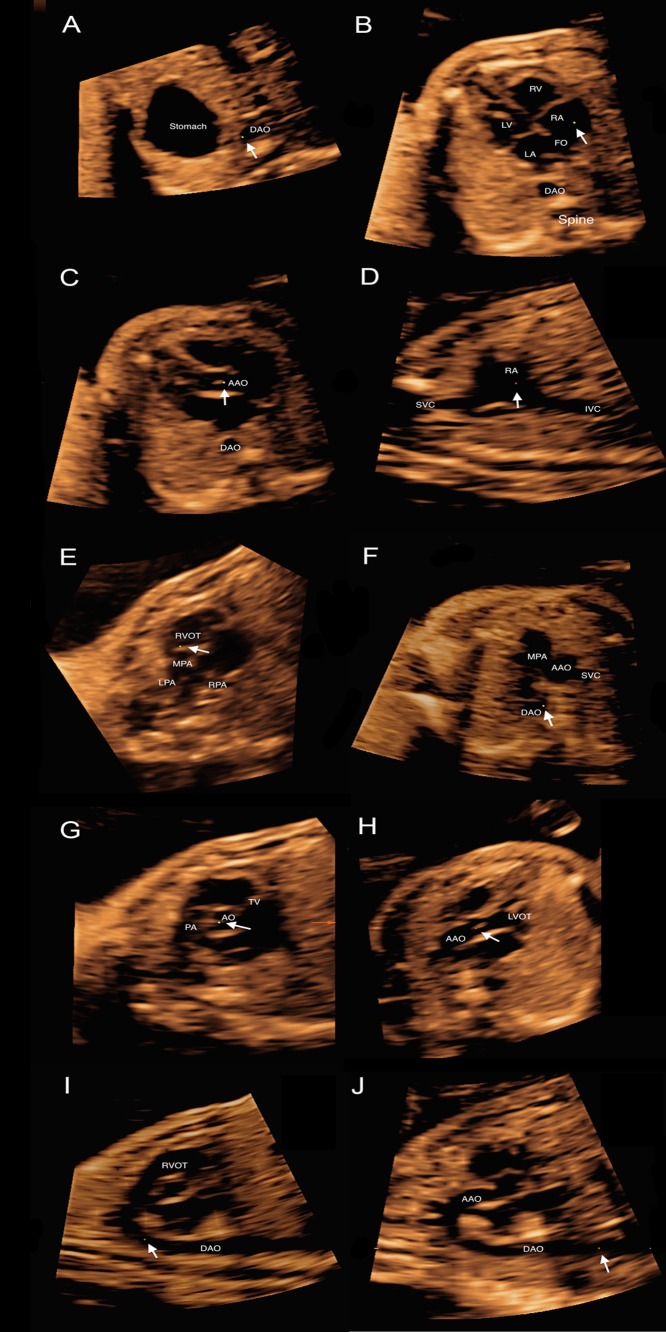
Required reconstructed fetal cardiac views: (A) abdominal view; (B) 4CV; (C) 5CV; (D) SVC + IVC; (E) RVOT; (F) 3VV; (G) short-axis view of great vessels; (H) LVOT; (I) DA; and (J) AA. DAO, descending aorta; LV, left ventricle; RV, right ventricle; LA, left atrium; RA, right atrium; FO, foramen ovale; AAO, ascending aorta; SVC, superior vena cava; IVC, inferior vena cava; RVOT, right ventricular outflow tract; MPA, main pulmonary artery; RPA, right pulmonary artery; LPA, left pulmonary artery; AO, aorta; AV, aortic valve; PA, pulmonary artery; TV, tricuspid valve; LVOT, left ventricular outflow tract; 4CV, four-chamber view; 3VV: three-vessel trachea view; DA, ductal arch; AA, aortic arch.

The required planes and scores were acquired using the multiplanar mode, and the steps to accomplish the scans were replayed as follows (4CV as the original plane). The cine loops were played in slow motion (50% speed routinely) and stopped at any time. First, every image was adjusted to make the apex of the heart be placed on the top left in plane A, with the DAO horizontal in plane B and straight in plane C (Fig 1). Secondly, the reference point was put in the DAO; the fetal heart was evaluated from inferior to superior; and the abdominal view, 4CV, 5CV, RVOT, and 3VV in plane A were checked by dragging the front-back key. Meanwhile, the z-axis was rotated in plane A, and the long-axis views of the ductal arch (DA) and AA in plane B were checked. When 4CV was displayed in plane A, the reference point was put in the right atrium (RA) and the z-axis was properly rotated to see the superior vena cava (SVC) and inferior vena cava (IVC) in plane B. When the RVOT view was seen in plane A, the reference point was put in the pulmonary valve (PV) and the y-axis was rotated to obtain a short-axis view of the great vessels. Additionally, when the reference point was put in the aortic valve in this view, the LVOT view appeared in plane B (Fig 2).

A successfully acquired STIC volume was defined as having acceptable displays of the 4CV, LVOT, RVOT, and 3VV, which are basic views in a regular obstetric cardiac screening. The scoring system is shown in Table 1 according to the demands for the prenatal diagnosis of CHD. A total of 8 points were counted in all in the 4CV, in which 3 points were obtained by checking the structures of the four chambers, 2 points for the connections of the interventricular septum (IVS) and atrial-ventricular valves (AVV) (clearly recognizing defects also gain a score), and 3 additional points assigned to the recognition of the opening of the AVV, foramen ovale (FO), and at least one pulmonary vein. A total of 4 points were counted in both the LVOT and RVOT, in which 3 points were obtained for the connections of the great vessels to appropriate ventricles and 1 point for the opening of the valves. The bifurcation of the pulmonary artery and the relationship between the great vessels both had 1 point in the short-axis view of the great vessels. The 3VV had 2 scores in all, and 1 point for recognition of the rough contours of three vessels and 2 points for clear contours and the relationship between the three vessels and the trachea. Connections of the SVC and IVC to the RA count as 1 score each. The continuity of the transverse AA to the longitudinal DAO and the ductus connected to the longitudinal aorta were the basic requirements for these two views (long-axis views of the AA and DA) and count as 2 points; 1 point was counted for the branches of the AA. One point was given for the abdominal view, and the location of the stomach bubble or hepatic veins was the criteria for awarding the point (Table 1).

**Table 1:** Scoring system of the volume data quality.

	Reconstructed structures	Scores in detail	Total scores
4CV	Structure of four chambers	3	8
	Connection of AVV and IVS	2	
	Opening of FO	1	
	Opening of AVV	1	
	One PV or more	1	
LVOT	Connection between great vessels and left ventricle	3	4
	Opening of AV	1	
RVOT	Connection between great vessel and right ventricle	3	4
	Opening of PV	1	
3VV	Rough contours	1	2
	Clear contours and definite relationship	2	
Short-axis view of the great vessels	Relationship of the great vessels	1	2
	PA bifurcation	1	
Long-axis view of the AA	Continuity	2	3
	Branches	1	
Long-axis view of the DA	Continuity	2	2
Long-cava view	Connection of both VCs	2	2
Abdominal view	Location of the stomach bubble or hepatic veins	1	1

4CV, Four-chamber view; LVOT, left ventricular outflow tract; RVOT, right ventricular outflow tract; 3VV, three-vessel view; AA, aortic arch; DA, ductal arch; AVV, atrioventricular valve; IVS, interventricular septum; FO, foramen ovale; PV, pulmonary valve; AO, aorta; AV, aortic valve; PA, pulmonary artery; VC, vena cava.

Possible influencing parameters such as age, pre-gestational BMI, GA, volume numbers, shadowing, extent of fetal movements, original views included, location of the spine, acquisition time, volume angle minus GA, and ROI setting were included in the statistical analysis. A *t* test was performed to compare the means, and a chi-square test was performed to compare proportions between the success and failure groups. Significance was defined as a *P* value <0.05. After the single-factor statistics were computed, the significant factors were put into logistic regression. The SPSS version 22.0 software (IBM, USA) was used for all statistical analysis.

## Results

During the study period (November 2014–January 2015), 452 fetuses were enrolled according to the inclusion criteria. The attempts of STIC acquisition were possible in all cases in this study. After data evaluation, 353 cases were scored as successes and 99 were identified as failures for the screening of fetal hearts. The acquisition rate of STIC was 100% and the success rate of qualified images after evaluation was 78.1%. The mean time of acquisition required was 4.44 minutes, ranged from 1 minute to 18 minutes, and the median (quartiles) time was 4 (3; 5) minutes. There were no significant differences in the time for acquisition among three centers (Table 2). The mean score and rate of qualified images in Center C was significantly lower than the two centers that received centralized training (*P* <0.05). Considering the notable difference, possible influence factors were compared among the three centers, and no significant differences were found in age, pre-gestational BMI and shadowing. Fetal movements and shadowing were unavoidable during fetal ultrasonography, both accounting for around 70% in each center. Between the two sufficient trained centers, Center B had a higher proportion of adequate ROI (*P* <0.05) and better spine location (*P* <0.05) than center A. The mean GA in center C (*P* <0.05) was significantly larger than those of center A and center B. Center C had a higher ratio (*P* <0.05) of choosing the mono original view to acquire STIC data, and the volume angle chosen was significantly smaller than those of the other two centers (*P* <0.05).

**Table 2:** Maternal and data characteristics among the three centers.

	Center A	Center B	Center C	P1	P2	P3
Case numbers	33	306	113	-	-	-
Sonographers	1	2	1	-	-	-
Time required (min)	4.15 ± 1.73	4.59 ± 2.52	4.12 ± 2.34	0.451	0.95	0.272
Age (year)	30.45 ± 3.83	29.86 ± 3.60	29.42 ± 3.39	0.362	0.148	0.285
GA (week)	22.72 ± 3.06	22.46 ± 2.42	25.08 ± 4.84	0.668	<0.05	<0.05
Pre-gestational BMI	20.55 ± 2.07	21.05 ± 2.69	20.9 ± 2.57	0.339	0.533	0.609
Mono original plane	24.2% (8/33)	30.4% (93/306)	92.9% (105/113)	0.463	<0.05	<0.05
Volume angle minus GA	9.63 ± 4.50	7.32 ± 2.64	4.06 ± 4.23	<0.05	<0.05	<0.05
Fetal movements	78.8% (26/33)	71.0% (217/306)	79.7% (90/113)	0.626	0.287	<0.05
Shadowing	72.7% (24/33)	69.6% (213/306)	77.9% (88/113)	0.710	0.538	0.095
Inadequate ROI	54.5% (18/33)	6.2% (19/306)	37.2% (42/113)	<0.05	0.074	<0.05
Spine location at 10–2 o’clock	9.1% (3/33)	1.6% (5/306)	12.4% (14/113)	<0.05	0.509	<0.05
Success rate	90.9% (30/33)	87.9% (269/306)	47.8% (54/113)	0.781	<0.05	<0.05
Mean score	21.73 ± 4.75	20.75 ± 3.95	15.19 ± 6.58	0.264	<0.05	<0.05

GA, Gestational age; BMI, body mass index [BMI = weight (kg)/height^2^ (m)]; ROI, region of interest; P1: P value between center A and B; P2: P value between center A and C; P3: P value between center B and C.

A total of 86.5% of the STIC volumes received more than 5 points in the scoring of 4CV, and the mean score was 5.83. The left and right ventricle outflow tract views were qualified as recognizing the connections of the great vessels and ventricles, and the rates were 92.5% and 92.7%, respectively. More than 1 point meant acceptable in the 3VV, short-axis view of the great vessels, long-axis view of the DA, long-cava view, and abdominal view; the rates were 89.8%, 81%, 81.4%, 80.1%, and 86.9%, respectively. The cutoff point was 2 in the long-axis view of the AA, and the rate was 63.9% (Table 3)(for detailed scores see S1 Table).

**Table 3:** Success rates of reconstructed cardiac views.

Cardiac views	Qualified score	Mean score	Success rate (%)	Full score rate (%)
4CV	5	5.83	86.5	12.2
LVOT	3	3.0	92.5	21.7
RVOT	3	2.98	92.7	18.6
3VV	1	1.36	89.9	45.1
Short-axis view of the great vessels	1	1.23	81	40.9
Long-axis view of the AA	2	1.59	63.9	16.4
Long-axis view of the DA	1	1.4	81.4	58.4
Long-cava view	1	1.15	80.1	35.2
Abdominal view	1	0.87	86.9	86.9

4CV, Four-chamber view; LVOT, left ventricular outflow tract; RVOT, right ventricular outflow tract; 3VV, three-vessel view; AA, aortic arch; DA, ductal arch.

The factors and parameters were analyzed and are listed in Table 4. Age, pre-gestational BMI, STIC volume numbers, and acquisition time were not different between the success group and the failure group. The GW, original cardiac views included, value of GW subtracted from volume angle, fetal movements, shadowing of bones, location of the spine, and ROI were shown to impact the quality of the STIC volume data. The missing GW data had almost no influence on the success and failure groups (the proportions were 3.7% and 4.0%, respectively).

**Table 4:** Single-factor analysis.

Parameters		Success	Failure	*P* value
	%/mean ± 2SD	*N* = 353	*N* = 99	
Age		29.86 ± 3.63	29.58 ± 3.3.4	0.510
Pre-gestational BMI		20.94 ± 2.64	21.14 ± 2.55	0.520
GW	20–23+6	83.0% (293/353)	64.6% (64/99)	<0.05
	24–27+6	7.6% (27/353)	6.1% (6/99)	
	≥28	5.7% (20/353)	25.3% (25/99)	
	Missing	3.7% (13/353)	4.0% (4/99)	
Volume numbers		3.61 ± 1.12	3.57 ± 1.36	0.730
Original view	4CV	39.1% (138/353)	68.7% (68/99)	<0.05
	More views	60.9% (215/353)	31.3% (31/99)	
Acquisition time	7.5/10 s	43.6% (154/353)	53.5% (53/99)	0.080
	12.5/15 s	56.4% (199/353)	46.5% (46/99)	
Volume angle minus GW	≥10°	6.8% (24/353)	5.1% (5/99)	<0.05
	5–9°	71.4% (252/353)	49.5% (49/99)	
	0–4°	15.3% (54/353)	32.3% (32/99)	
	<0°	2.8% (10/353)	9.1% (9/99)	
	Missing	3.7% (13/353)	4.0% (4/99)	
Fetal movements	None	29.2% (103/353)	16.2% (16/99)	<0.05
	Occasionally	58.9% (208/353)	57.6% (57/99)	
	Frequently	11.9% (42/353)	26.3% (26/99)	
Shadowing	None	32% (113/353)	14.1% (14/99)	<0.05
	Exist	68% (240/353)	85.9% (85/99)	
Spine location	4–8 o’clock	87% (307/353)	60.6% (60/99)	<0.05
	2–4/8–10 o’clock	11.9% (42/353)	21.2% (21/99)	
	10–2 o’clock	1.1% (4/353)	18.2% (18/99)	
ROI	Adequate	86.7% (306/353)	67.7% (67/99)	<0.05
	Inadequate	13.3% (47/353)	32.3% (32/99)	

BMI, Body mass index [BMI = weight (kg)/height^2^ (m)]; GW, gestational week; ROI, region of interest; 4CV, four-chamber view.

**Fig 3:**
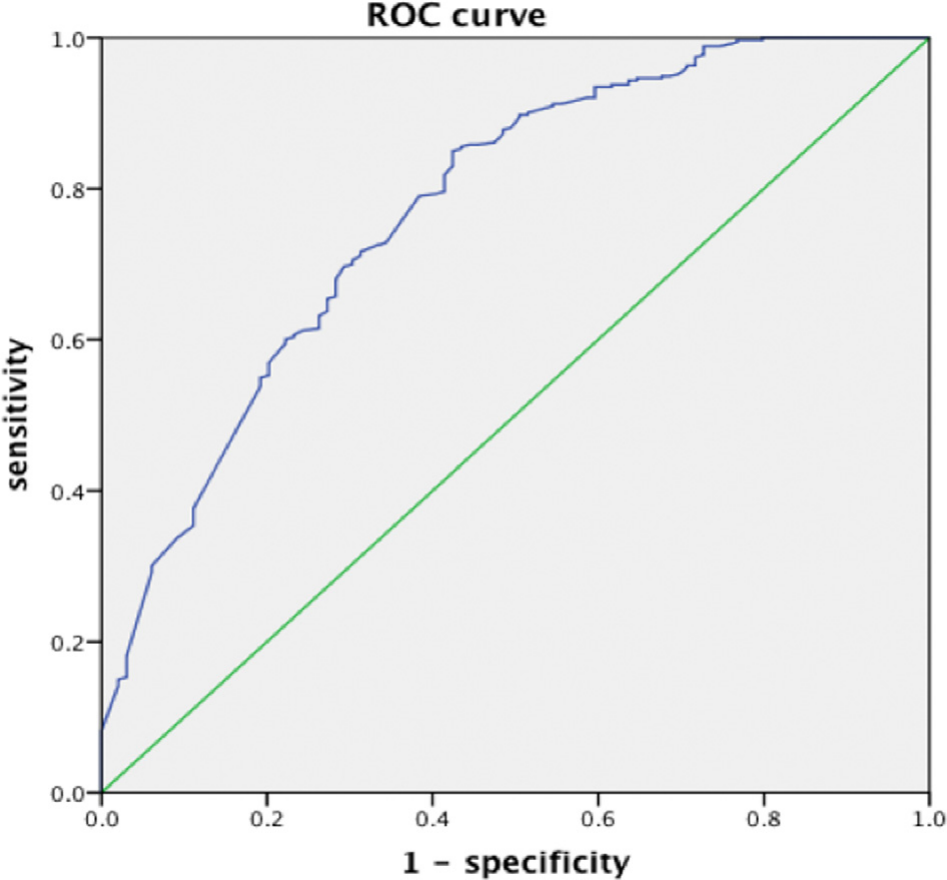
ROC curve for predicting the influence factor of STIC quality.

**Fig 4:**
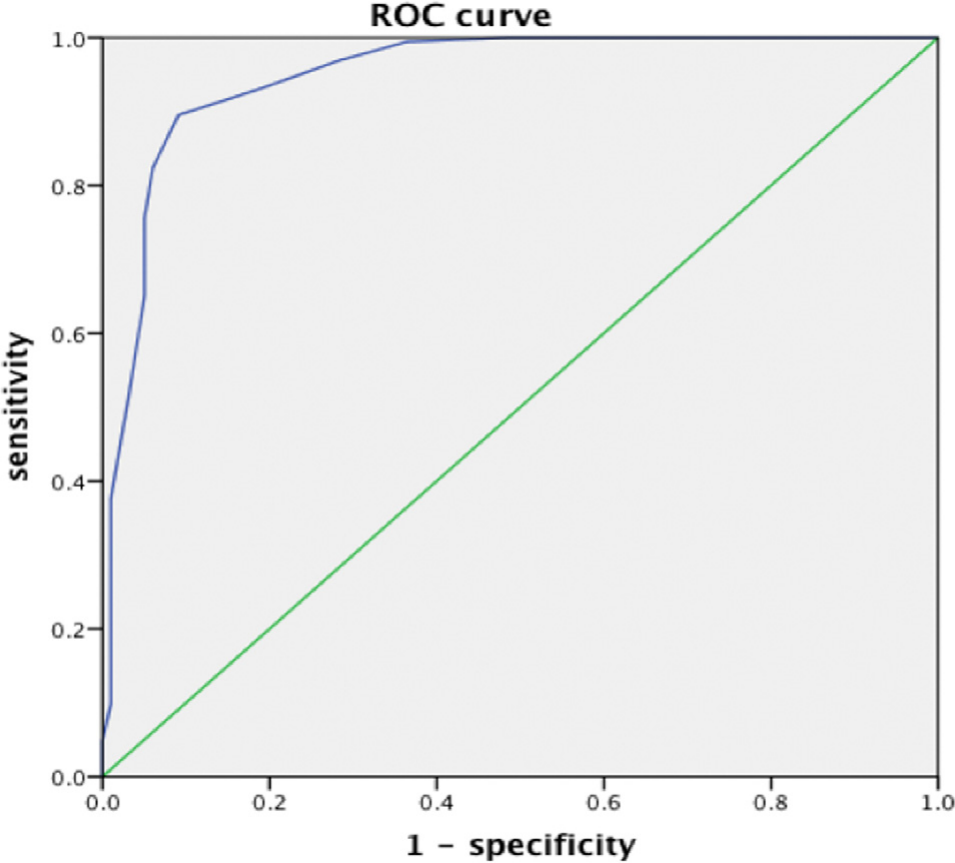
ROC curve for aggregate score criteria and stated obstetric screening plane criteria (used in this study) for judging the STIC quality.

The results of the binary logistic regression analysis of the significant influence factors revealed that frequent fetal movements (*P* = 0.022), shadowing (*P* = 0.004), spine location at 10–2 o’clock (*P* = 0.000), and large GW (>28 W, *P* = 0.035) significantly reduced the success rate of STIC acquisition. More original views (*P* = 0.019), 4CV incorporated with other views, such as 3VV and AA, advanced the overall quality of fetal cardiac STIC (Table 5). The area under the receiver operating characteristic (ROC) curve was 0.775 (Fig 3), demonstrating that the obtained influence factors predicted well whether a satisfactory STIC was obtained. All cardiac view scores were not used for assessing the qualified STIC data in this study, but the total scores were consistent with the standard that was used, and the area under the ROC curve was 0.949 (Fig 4).

**Table 5:** Results of binary logistic regression.

Parameters		OR (95% CI)	*P* value
Fetal movements	None	1	
	Occasionally	0.57 (0.29, 1.12)	0.102
	Frequently	0.37 (0.16, 0.87)	0.022
Shadowing	None	1	
	Exist	0.36 (0.19, 0.72)	0.004
Location of spine	4–8 o’clock	1	
	2–4/8–10 o’clock	0.56 (0.29, 1.08)	0.083
	10–2 o’clock	0.08 (0.02, 0.27)	0.000
GW	20 W–23 W+6D	1	
	24 W–27 W+6D	1.83 (0.63, 5.33)	0.268
	≥28 W	0.39 (0.16, 0.94)	0.035
Original view	4CV	1	
	More views	0.51 (0.25, 0.89)	0.019
ROI	Adequate	1	
	Inadequate	0.67 (0.35, 1.29)	0.229
Volume angle minus GW	≥10°	1	
	5–9°	0.74 (0.22, 2.51)	0.627
	0–4°	0.37 (0.10, 1.35)	0.132
	<0°	0.79 (0.14, 4.43)	0.789

GW, Gestational week; ROI, region of interest; 4CV, four-chamber view; W, weeks; D, days.

## Discussion

As claimed in the 2014 American Heart Association guidelines for the components of fetal echocardiograms, fetal echocardiograms should contain 2D imaging, rhythm assessments, color flow map imaging, pulsed Doppler interrogation, continuous-wave Doppler, and ventricular function parameters [13]. A 4D ultrasound with STIC has its own advantages but cannot be a single tool for prenatal diagnosis. Several studies have proved its potential as a screening tool for CHDs and its added diagnostic value in certain types of CHD [14–15]. A meta-analysis on different scan protocols of fetal echocardiography demonstrated that STIC has similar sensitivity to that of ECEE (extend cardiac echography examination) and 4CV + OTV + 3VV scan protocols but has a lower specificity than other tests [16]. The present multicenter study obtained a 78.1% success rate and a success rate of approximately 90% at the two sufficiently trained centers. The reason why center C had an unsatisfactory success rate may mostly be due to its absence from the training. As a result, the study concluded that the success rate of STIC was considerably better after efficient training.

The satisfactory acquisition of STIC has been shown in several studies, and the success rate varied from one study to another. When STIC was first introduced in 2003, Vinals *et al* reported a 94.2% success rate using the STIC sweep alone and a 96.2% success rate after adding a 3D-multiplanar assessment [9]. Uittenbogaard *et al* reported a 75.7% (112/148) rate of successful STIC volume acquisition and 64.8% rate of STIC volume acquisitions of high or sufficient quality [14]. Different criteria have achieved different success rates of STIC volume acquisition. In the present study, the acquisition was possible in all enrolled fetuses and all attempts were sent to our heart center for analysis. The cutoff point used to judge whether an STIC volume was successfully for evaluation was the ability to perform prenatal screening for CHDs, which means that essential structures must be seen in the STIC volume data. In consideration of the segmental diagnosis of major CHDs, nine fetal cardiac views were taken in this study, including four basic views in a regular obstetric screening, as a scoring system. To value the screening efficiency, these data were compared with those of the four basic views in a regular obstetric scan. Using the total scores of each volume as another assessment standard was also considered, and this approach was found to have acceptable consistency.

The four-chamber view was almost the only original view used when acquiring STIC volumes in previous studies [17–18], and some studies showed that choosing the transverse plane as the original plane could achieve high-quality views of 4CV, 5CV, and 3VV, whereas the sagittal plane could achieve a better view of the AA, DA, and VC [19]. The present study considered more views, including 4CV as an original plane, and ultimately found that more views in the STIC in one fetus gave more information. The AA view had a lower success rate compared with other views due to its low usage rate as an original plane. The success rate of each plane was satisfactory, whereas the full score rate was significantly lower than the cutoff scores; for example, awarding the cutoff point (5 points) of 4CV accounted for 86.5% while awarding the full 8 points only accounted for 12.2% (Table 3), similar to other reconstructed views. According to this result, detailed accurate diagnosis of fetal CHD may not be sufficient via STIC technology alone. But this study found the STIC technology as a huge potential in screening for major CHDs.

The fact that the GW range, fetal situation at the time, and machine parameters chosen all influence the successful acquisition of qualified STIC may restrict its widespread use. After reviewing the STIC data from 452 fetuses (almost 1600 volumes), the current study found that the GW, original cardiac view included, value of volume angle minus GA, fetal movements, shadowing, location of the spine, and ROI had impact on the quality of the STIC volume data. The logistic regression results showed that frequent fetal movements, shadowing, spine location at 10–2 o’clock, and large GW (>28 weeks) significantly reduced the success rate of qualified STIC acquisition. In the presence of any of these conditions, STIC sonography may not provide more useful information compared with the conventional 2D sonography, since both are affected by the aforementioned conditions. For other conditions, STIC will become an optional screening tool for CHDs for obstetric sonographers inexperienced in fetal echocardiography. As a result, an optimal acquisition scheme for STIC should consist of following aspects: (1) sufficient training on operators; (2) appropriate gestational age: no more than 28 GW; (3) suitable fetal conditions: avoiding frequent fetal movements, shadowing and spine location at 10–2 o’clock; (4) parameter setting: adequate ROI, volume angle 5–9° over the value of GW and choosing more original views including 4CV.

In conclusion, the present study arranged a multicenter obstetric–cardiology connected mode by the acquisition and offline analysis of the STIC volume data. The study determined the factors that affected the successful acquisition of qualified STIC volumes, the conditions in which qualified STIC volume data will be obtained, the parameters that are the most applicable and thus established an optimal acquisition scheme. Fetal cardiac-STIC seems a feasible tool for prenatal screening of congenital heart diseases. The standard acquisition scheme, transmission, and offline analysis of the STIC volume data are believed to open up new opportunities for remote consultation and improvement in the prenatal diagnosis of fetal CHDs.

## Supporting Information

S1 Table Raw data of basic characteristics of enrolled pregnancies and scores of STIC data. (XLSX)
